# Ultraprocessed Food Consumption and Cardiometabolic Risk Factors in Children

**DOI:** 10.1001/jamanetworkopen.2024.11852

**Published:** 2024-05-17

**Authors:** Nadine Khoury, María Ángeles Martínez, Tany E. Garcidueñas-Fimbres, Belén Pastor-Villaescusa, Rosaura Leis, Sara de Las Heras-Delgado, María L. Miguel-Berges, Santiago Navas-Carretero, Olga Portoles, Karla Alejandra Pérez-Vega, Jose Manuel Jurado-Castro, Rocío Vázquez-Cobela, Gisela Mimbrero, Raquel Andía Horno, J. Alfredo Martínez, Katherine Flores-Rojas, Rosaura Picáns-Leis, Verónica Luque, Luis A. Moreno, Cristina Castro-Collado, Mercedes Gil-Campos, Jordi Salas-Salvadó, Nancy Babio

**Affiliations:** 1 Universitat Rovira i Virgili Departament de Bioquímica i Biotecnologia, Unitat de Nutrició Humana, ANUT-DSM group, Spain; 2 Institut d’Investigació Sanitària Pere Virgili, Reus, Spain; 3 Consorcio Centro de Investigación Biomédica en Red, M. P. Fisiopatología de la Obesidad y Nutrición, Instituto de Salud Carlos III, Madrid, Spain; 4 Metabolism and Investigation Unit, Maimónides Institute of Biomedicine Research of Córdoba, Reina Sofia University Hospital, University of Córdoba, Córdoba, Spain; 5 Primary Care Interventions to Prevent Maternal and Child Chronic Diseases of Perinatal and Developmental Origin, Instituto de Salud Carlos III, Madrid, Spain; 6 Unit of Pediatric Gastroenterology, Hepatology and Nutrition, Pediatric Service, Hospital Clínico Universitario de Santiago, Santiago de Compostela, Spain; 7 Pediatric Nutrition Research Group, Health Research Institute of Santiago de Compostela, Unit of Investigation in Nutrition, Growth and Human Development of Galicia-Universidad de Santiago de Compostela, Santiago de Compostela, Spain; 8 Growth, Exercise, Nutrition and Development Research Group, University of Zaragoza, Spain; 9 Instituto Agroalimentario de Aragón, University of Zaragoza, Spain; 10 Instituto de Investigación Sanitaria de Aragón, Zaragoza, Spain; 11 Center for Nutrition Research, Faculty of Pharmacy and Nutrition, Department of Nutrition, Food Science and Physiology, University of Navarra, Pamplona, Spain; 12 Navarra Medical Research Institute, Pamplona, Spain; 13 Department of Preventive Medicine and Public Health, University of Valencia, Spain; 14 Hospital del Mar Medical Research Institute, Barcelona, Spain; 15 Centre d’Atenció Primària, Institut Català de la Salut, Reus, Spain; 16 Pediatrics, Nutrition, and Development Research Unit, Universitat Rovira I Virgili, Reus, Spain

## Abstract

**Importance:**

High intake of ultraprocessed foods (UPFs) has been associated with higher cardiometabolic risk in adults; however, the evidence in children is limited.

**Objective:**

To investigate the association between UPF consumption and cardiometabolic risk factors in the Childhood Obesity Risk Assessment Longitudinal Study (CORALS).

**Design, Setting, and Participants:**

This baseline cross-sectional analysis was conducted using the data of CORALS participants recruited between March 22, 2019, and June 30, 2022. Preschool children (aged 3-6 years) were recruited from schools and centers in 7 cities in Spain. Inclusion criteria included informed consent signed by parents or caregivers and having a completed a set of questionnaires about the child’s prenatal history at home. Exclusion criteria included low command of Spanish or unstable residence.

**Exposure:**

Energy-adjusted UPF consumption (in grams per day) from food frequency questionnaires and based on the NOVA food classification system.

**Main Outcomes and Measures:**

Age- and sex-specific *z* scores of adiposity parameters (body mass index [BMI], fat mass index, waist-to-height ratio, and waist circumference) and cardiometabolic parameters (diastolic and systolic blood pressure, fasting plasma glucose, homeostasis model assessment for insulin resistance, high-density and low-density lipoprotein cholesterol, and triglycerides) were estimated using linear regression models.

**Results:**

Of 1509 enrolled CORALS participants, 1426 (mean [SD] age, 5.8 [1.1] years; 698 boys [49.0%]) were included in this study. Mothers of children with high UPF consumption were younger, had a higher BMI, were more likely to have overweight or obesity, and had lower education levels and employment rates. Compared with participants in the lowest tertile of energy-adjusted UPF consumption, those in the highest tertile showed higher *z* scores of BMI (β coefficient, 0.20; 95% CI, 0.05-0.35), waist circumference (β coefficient, 0.20; 95% CI, 0.05-0.35), fat mass index (β coefficient, 0.17; 95% CI, 0.00-0.32), and fasting plasma glucose (β coefficient, 0.22; 95% CI, 0.06-0.37) and lower *z* scores for HDL cholesterol (β coefficient, −0.19; 95% CI, −0.36 to −0.02). One-SD increments in energy-adjusted UPF consumption were associated with higher *z* scores for BMI (β coefficient, 0.11; 95% CI, 0.05-0.17), waist circumference (β coefficient, 0.09; 95% CI, 0.02-0.15), fat mass index (β coefficient, 0.11; 95% CI, 0.04-1.18), and fasting plasma glucose (β coefficient, 0.10; 95% CI, 0.03-0.17) and lower HDL cholesterol (β coefficient, −0.07; 95% CI, −0.15 to −0.00). Substituting 100 g of UPFs with 100 g of unprocessed or minimally processed foods was associated with lower *z* scores of BMI (β coefficient, −0.03; 95% CI, −0.06 to −0.01), fat mass index (β coefficient, −0.03; 95% CI, −0.06 to 0.00), and fasting plasma glucose (β coefficient, −0.04; 95% CI, −0.07 to −0.01).

**Conclusions and Relevance:**

These findings suggest that high UPF consumption in young children is associated with adiposity and other cardiometabolic risk factors, highlighting the need for public health initiatives to promote the replacement of UPFs with unprocessed or minimally processed foods.

## Key Points

**Question:**

What is the association of consuming ultraprocessed foods (UPFs) with cardiometabolic risk factors in children?

**Findings:**

In this cross-sectional study of 1426 children, higher consumption of UPFs was positively associated with body mass index, waist circumference, fat mass index, and fasting plasma glucose and negatively associated with high-density lipoprotein cholesterol concentrations.

**Meaning:**

These findings highlight the need for public health initiatives to promote the replacement of UPFs with unprocessed or minimally processed foods.

## Teaser

This cross-sectional study investigates the association between ultraprocessed food consumption and cardiometabolic risk factors in preschool-aged children in Spain.

## Introduction

The presence of abnormal cardiometabolic risk factors often begins in childhood, highlighting the importance of identifying and controlling them early to delay or prevent cardiovascular disease (CVD) in the future.^1^ Modifiable risk factors (eg, diet and physical activity) may contribute to the development of recognized cardiometabolic risk factors.^2,3,4^ Emerging studies have shed light on the potential role of ultraprocessed foods (UPFs) in determining the risk of chronic diseases,^5,6,7^ independent of their nutritional profiles.^8^

Commonly, UPFs represent a category of food products that undergo extensive industrial processing, often containing multiple ingredients, additives, and preservatives to make them not only convenient (ready to eat) but also palatable and appealing. This approach has been used to create the most widely used UPF classification, the NOVA Food Classification system.^9,10^ Ultraprocessed foods are typically rich in saturated fats, sugars, sodium, and other substances (eg, additives) and lower in essential nutrients, all of which are associated with cardiometabolic health.^11,12^ Due to their high availability and affordability and wide marketing to children, UPFs have become increasingly frequent in modern diets, particularly among children, adolescents,^13,14,15^ and their families, and especially among individuals and families with low socioeconomic status and educational levels in which obesity is more prevalent.^16^ Additionally, the habits established during early childhood often track to later ages^17^ and carry into adulthood, compounding the risk of CVD and other noncommunicable diseases.^18,19,20^

Previous observational studies in adults have reported positive associations between UPF consumption and obesity,^21^ type 2 diabetes,^22^ CVD,^23^ and all-cause mortality^24^; however, the epidemiologic evidence in children remains limited and controversial.^25^ While the majority of studies have reported unfavorable associations with body mass index (BMI), others did not find this association, and few have focused on cardiometabolic risk factors.^25^

Given the public health burden of CVDs and the increasing availability of UPFs, having a better understanding of potential associations between UPF consumption and cardiometabolic risk factors in children is essential. Therefore, the aim of this study was to examine the associations between UPF consumption and cardiometabolic risk factors in a population of Spanish preschool children (aged 3-6 years).

## Methodology

### Population and Study Design

This cross-sectional study was conducted using data from the Childhood Obesity Risk Assessment Longitudinal Study (CORALS), which followed the Strengthening the Reporting of Observational Studies in Epidemiology (STROBE) reporting guideline. The ethics committee of each of recruitment center approved the study protocol, which was conducted following the standards of the Declaration of Helsinki.^26^ Parents or caregivers provided written informed consent.

CORALS is an ongoing prospective multicenter study conducted in 7 Spanish centers aiming to identify potential risk factors for childhood obesity over a 10-year follow-up period. A detailed description of the CORALS is published elsewhere.^27^ Between March 22, 2019, and June 30, 2022, eligible participants aged 3 to 6 years at enrollment were recruited from schools across 7 cities in Spain. To be enrolled in the study, parents or caregivers had to sign a consent form, attend the inclusion face-to-face visit and complete several questionnaires at home for data collection on leisure time physical activity, 3-day food consumption, and sociodemographics. The exclusion criteria included belonging to a family with difficulty collaborating due to low command of Spanish or unstable residence.

### Dietary Intake of UPFs and NOVA Food Classification System

To estimate the dietary intake of UPFs, trained dietitians (B.P.-V., S.d.L.H.D., M.L.M.-B., K.A.P.-V., and R.V.-C.) used the validated, semiquantitative, 125-item food and beverage frequency COME-Kids questionnaire.^28^ Participants with energy intake below the first percentile or above the 99th percentile were excluded to minimize misreporting (details are provided in the eAppendix in Supplement 1). The NOVA Food Classification system was used to determine the consumption of food depending on its degree of processing^27^ (details are provided in the eAppendix and eTable 1 in Supplement 1).

### Outcomes

#### Adiposity Measurements

Adiposity measurements and cardiometabolic risk factor assessments were conducted in health care centers. Weight and body fat mass were measured using a precision scale and an octopolar multifrequency bioelectrical impedance device (MC780MAS; Tanita). Height was measured using a portable seca 213 stadiometer according to standard procedures. Body mass index was calculated and categorized as underweight or normal weight or as overweight or obesity based on pediatric cutoffs.^29^ Waist circumference was determined using a flexible, nonextensible measuring tape. The fat mass index was estimated by dividing body fat mass in kilograms by height in meters squared.^30^ Waist-to-height ratio was estimated by dividing waist circumference in centimeters by height in centimeters.^31^

#### Cardiometabolic Risk Factors Assessment

Blood pressure was measured in the nondominant arm 3 times, with a 5-minute gap between each measurement, using an automatic oscillometer (M3 Intelligence HEM-75051-EV; OMRON Healthcare) equipped with a child-sized cuff. Eight-hour fasting blood samples were collected from participants, and serum total cholesterol, high-density lipoprotein (HDL) cholesterol, low-density lipoprotein (LDL) cholesterol, triglycerides, plasma glucose, and insulin concentrations were measured using standard procedures. Homeostasis model assessment for insulin resistance (HOMA-IR) was calculated as fasting insulin (μIU/mL) × fasting glucose (mmol/L) / 22.5.

### Covariates

Parents or caregivers were provided with a set of questionnaires to complete at home, gathering information on early life factors, maternal characteristics, and lifestyle patterns. To assess physical activity, the total time (in hours) of engagement in sports and physical activities per week was estimated using a previously validated questionnaire.^32^ An 18-item questionnaire for children was used to assess adherence to the Mediterranean diet, an indicator of diet quality.^27^

### Statistical Analysis

The current analysis was conducted using the CORALS database updated through January 20, 2023. Analysis of descriptive baseline characteristics are reported as mean (SD) or median (IQR) for continuous variables and as numbers with percentages for categorical variables using one-way analysis of variance and χ^2^ test, respectively.

Consumption of UPFs (in grams per day) was adjusted for total energy intake using the residual regression method.^33^ Intake of UPFs in grams per day was calculated instead of energy percentage to account for foods with no energy content (eg, artificially sweetened beverages) and for nonnutritional concerns associated with food processing (eg, food additives). Participants were categorized by tertiles of energy-adjusted UPF consumption, ranging from tertile 1 for the lowest intake to tertile 3 for the highest intake.

Age- and sex-specific *z* scores of each outcome were estimated from standardized residuals conducted using linear regression models. Missing data of less than 5% for covariates were imputed to the mean and to the highest frequency category for quantitative and qualitative confounders, respectively.^34^ Multivariable linear regression models were fitted to assess the associations (β coefficient and 95% CI) between tertiles of energy-adjusted UPFs and *z* scores of cardiometabolic risk factors. The first tertile (lowest intake) was considered the reference. Models were adjusted for maternal education level (primary or lower, secondary or university), maternal BMI (underweight, normal overweight, obesity), physical activity (minutes per week), exclusive breastfeeding (yes or no), recruitment center size (<200, 200-400, >400 participants), and NOVA group 1, 2, or 3 (as detailed in eTable 1 in Supplement 1). To assess the linear trend, the median value of each tertile of UPF consumption was modeled as a continuous variable. The analysis was also conducted in a continuous form, with a 1-SD increment and using the same confounders.

Additionally, a simulation model was fitted to substitute 100 g of consumed UPFs with 100 g of unprocessed or minimally processed food to examine the association of healthier food consumption with the outcomes. The theoretical impact of substituting 1 food group for another was assessed by introducing both variables simultaneously as continuous variables into the model. Differences in the β coefficients, variances, and covariance were used to estimate the β coefficients and 95% CIs for the substitution association. Sensitivity analyses were conducted to assess associations according diet quality, maternal education, and socioprofessional level (details provided in the eAppendix in Supplement 1).

Data were analyzed using Stata, version 14 software (StataCorp LLC). All statistical tests were 2-sided, and *P* < .05 was deemed statistically significant.

## Results

A total of 1426 participants (mean [SD] age, of 5.8 [1.1] years; 698 boys [49.0%] and 728 girls [51.0%]) were included in this study after excluding 54 participants lacking the food and beverage frequency questionnaire and 29 with missing data or implausible reported energy intake (eFigure 1 in Supplement 1). The characteristics of the study population across tertiles of energy-adjusted UPF consumption are shown in Table 1. Children in the third tertile (highest UPF consumption) had a higher BMI *z* score, waist-to-height ratio, fat mass index, systolic blood pressure, and overweight or obesity prevalence and lower HDL and LDL cholesterol. Mothers whose children were categorized in the highest tertile of energy-adjusted UPF consumption were younger, had a higher BMI, were more prone to be living with overweight or obesity, were less likely to have exclusively breastfed their children, and had lower educational achievement and employment rates.

**Table 1.** General Characteristics of Study Participants Across Tertiles of Energy-Adjusted Ultraprocessed Food Consumption

Characteristic	Tertiles of energy-adjusted ultraprocessed food consumption				
	1 (Lowest) (n = 476)	2 (n = 475)	3 (Highest) (n = 475)	*P* value^a^
**Child**				
Age, mean (SD), y	4.8 (1.1)	5.0 (1.1)	5.2 (1.1)	<.001
Sex, No. (%)				
Girls	237 (49.8)	247 (52)	244 (51.4)	.78
Boys	239 (50.2)	228 (48)	231 (48.6)	
Adiposity, mean (SD)				
BMI	16.1 (1.85)	16.3 (2.02)	16.6 (2.21)	.002
BMI *z* score	−0.11 (0.91)	0.00 (1.01)	0.10 (1.07)	.01
Weight status, No. (%)				
Underweight or normal weight	381 (82.5)	370 (79.1)	351 (75.0)	.02
Overweight or obesity	81 (17.5)	98 (20.9)	115 (25.0)	
Waist circumference, mean (SD), cm	51.7 (5.93)	52.3 (6.41)	52.0 (7.43)	.43
Waist-to-height ratio, mean (SD)	0.48 (0.05)	0.48 (0.05)	0.47 (0.06)	.01
Fat mass index, mean (SD)	3.72 (1.11)	3.88 (1.25)	3.97 (1.31)	.02
**Lifestyle**				
Physical activity, mean (SD), min/wk	178 (110)	181 (111)	184 (121)	.69
Cardiometabolic risk factors				
Systolic blood pressure, mean (SD), mm Hg	102 (12.3)	103 (12.7)	104 (13.5)	.003
Diastolic blood pressure, mean (SD), mm Hg	63.9 (11.6)	65.2 (11.5)	64.3 (12.0)	.26
Fasting plasma glucose, mean (SD), mg/dL	76.8 (9.24)	77.6 (9.39)	77.0 (8.85)	.50
HDL cholesterol, median (IQR), mg/dL	58.0 (50.0-66.7)	58.0 (50.0-66.2)	55.0 (47.0-64.0)	.01
LDL cholesterol, median (IQR), mg/dL	96.0 (84.0-113)	96.0 (83.0-112)	93.0 (78.0-110)	.04
Triglycerides, median (IQR), mg/dL	51.0 (42.0-62.5)	51.5.0 (43.0-63.0)	56.0 (45.0-69.0)	.04
**Maternal**				
Age, mean (SD), y	39.5 (4.69)	38.7 (4.73)	37.6 (5.14)	<.001
BMI, mean (SD)	24.1 (4.25)	24.4 (4.46)	25.9 (5.49)	<.001
Exclusive breastfeeding, No. (%)	194 (40.8)	37.9 (180)	157 (33.1)	.046
Weight status				
Underweight or normal weight	322 (67.6)	310 (65.3)	273 (57.5)	.003
Overweight or obesity	154 (32.3)	165 (34.7)	202 (42.5)	
Educational level				
Primary or lower	24 (5.04)	39 (8.21)	72 (15.2)	<.001
Secondary	157 (32.9)	161 (33.9)	235 (49.5)	
University	281 (59.0)	263 (55.4)	155 (32.6)	
Not reported	14 (2.9)	13 (2.5)	13 (2.7)	
Socioprofessional category				
Homemaker, student, retired, or unemployed	122 (25.6)	115 (24.2)	163 (34.3)	.005
Employed	354 (74.4)	360 (75.8)	312 (65.7)	

Abbreviations: BMI, body mass index (as measured by weight in kilograms divided by height in meters squared); HDL, high-density lipoprotein; LDL, low-density lipoprotein.

SI conversions: To convert glucose to mmol/L, multiply by 0.0555; to convert HDL and LDL to mmol/L, multiply by 0.0259; to convert triglycerides to mmol/L, multiply by 0.0113.

^a^ *P* values for comparisons were tested by 1-way analysis of variance or χ^2^ test, as appropriate, according to tertiles of energy-adjusted ultraprocessed food consumption.

General dietary characteristics of participants are shown in Table 2. Children in the top tertile were more likely to consume higher amounts of total energy, carbohydrates, yogurt, other dairy products, sugar and candy, and sugary beverages and lower amounts of protein, fat, monounsaturated and polyunsaturated fatty acids, fiber, milk, cheese, white meat, unprocessed red meat, eggs, fish, seafood, vegetables, fruits, nuts, legumes, whole and refined cereals, and oils and fat.

**Table 2.** Baseline Dietary Characteristics of Participants Across Tertiles of Energy-Adjusted Ultraprocessed Food Consumption in the Diet

Characteristic	Tertiles of energy-adjusted ultraprocessed food consumption, mean (SD)			
	1 (Lowest) (n = 476)	2 (n = 475)	3 (Highest) (n = 475)	*P* value^a^
Energy-adjusted ultraprocessed food (n = 1426), g/d	192.8 (76.7)	354.8 (39.9)	593.4 (167)	<.001
Dietary intake contribution				
Total energy intake, kcal/d	1867.3 (420.1)	1626.4 (388.3)	1780.8 (470.7)	<.001
Carbohydrates, % of total energy intake	40.9 (5.7)	42.9 (4.9)	46.1 (5.1)	<.001
Proteins, % of total energy intake	14.9 (2.3)	15.1 (2.2)	14.9 (2.1)	.19
Protein intake per body weight, g/kg/d	3.8 (1.2)	3.2 (0.9)	3.3 (1.1)	<.001
Total fat, % of total energy intake	44.1 (6.4)	41.9 (5.1)	38.9 (5.1)	<.001
Saturated fatty acids, % of total energy intake	13.9 (2.3)	13.9 (2.0)	13.8 (2.0)	.71
Monounsaturated fatty acids, % of total energy intake	20.6 (4.8)	18.7 (3.8)	16.3 (3.5)	<.001
Polyunsaturated fatty acids, % of total energy intake	6.2 (1.7)	6.3 (1.6)	6.0 (1.8)	.01
Fiber, g/d	17.1 (5.3)	13.6 (3.8)	13.1 (3.7)	<.001
≥14 g/1000 kcal, No. (%)	43 (9.0)	12 (2.5)	3 (0.6)	<.001
Sodium, mg/d	138.8 (1.9)	138.5 (7.9)	139.3 (1.9)	.08
Dairy products, g/d				
Milk	384.3 (280.6)	298.2 (197.7)	286.8 (213/7)	<.001
Yogurt	92.7 (80.4)	96.5 (67.5)	151.7 (130.9)	<.001
Cheese	16.0 (17.5)	11.8 (10.6)	11.6 (14.9)	<.001
Other dairy products	50.1 (53.9)	70.9 (66.2)	155.4 (150.1)	<.001
Protein foods, g/d				
White meat	26.7 (14.9)	24.7 (9.81)	24.2 (13.3)	.01
Unprocessed red meat	22.3 (23.6)	19.9 (14.5)	18.1 (13.1)	.001
Processed and derivatives meat products	25.8 (16.6)	24.1 (13.9)	28.4 (19.0)	.001
Egg	26.7 (21.1)	23.7 (9.6)	22.5 (17.3)	<.001
Fish and seafood	36.0 (19.3)	33.4 (17.5)	33.1 (21.3)	.04
Vegetables and fruits, g/d				
Vegetables	101 (59.4)	68.3 (43.7)	58.8 (44.0)	<.001
Tubers	42.7 (22.8)	41.2 (19.0)	40.9 (20.8)	.35
Fruits	253.9 (160.4)	180.2 (105.9)	150.6 (108.5)	<.001
Nuts, g/d	5.1 (7.6)	3.2 (4.4)	2.7 (3.9)	<.001
Cereals and legumes, g/d				
Legumes	15.5 (11.9)	13.4 (6.9)	13.8 (7.3)	.001
Refined cereals	81.6 (44.2)	73.9 (34.8)	70.8 (35.7)	<.001
Whole cereals	14.1 (23.9)	6.59 (12.8)	4.65 (10.7)	<.001
Miscellaneous, g/d				
Oil and fats	34.5 (18.3)	24.4 (13.5)	19.7 (12.9)	<.001
Pastries	42.0 (36.9)	41.5 (33.4)	43.6 (32.5)	.60
Sugars and candies	6.2 (7.2)	5.7 (6.1)	6.7 (10.7)	.13
Beverages, mL/d				
Water	895.2 (350.5)	871.9 (385.2)	840.0 (377.7)	.07
Sugary beverages	92.8 (126.4)	81.1 (94.2)	192.0 (191.3)	<.001
Tea and infusions	6.7 (26.2)	7.6 (30.0)	6.2 (35.1)	.76

^a^ *P* values for comparisons were tested by one-way analysis of variance or χ^2^ test, as appropriate, according to energy-adjusted ultraprocessed food consumption.

Cross-sectional associations between energy-adjusted UPF consumption across tertiles and by 1-SD increment (in grams per day) and cardiometabolic risk factors are shown in Table 3. Compared with participants in the lowest tertile, those in the top tertile had higher *z* scores of waist circumference (β coefficient, 0.20; 95% CI, 0.05-0.35), BMI (β coefficient, 0.20; 95% CI, 0.05-0.35), fat mass index (β coefficient, 0.17; 95% CI, 0.00-0.32), and fasting plasma glucose (β coefficient, 0.22; 95% CI, 0.06-0.37). Additionally, participants in the highest tertile had a lower *z* score of HDL cholesterol (β coefficient, −0.19; 95% CI, −0.36 to −0.02). After adjusting for the Mediterranean diet score (12 points), the associations were maintained for the *z* scores of fasting plasma glucose (β coefficient, 0.17; 95% CI, 0.03-0.31) and HDL cholesterol (β coefficient, −0.20; 95% CI, −0.36 to −0.05) (eTable 2 in Supplement 1). Positive associations were also observed between 1-SD increments of UPF consumption and *z* scores of waist circumference (β coefficient, 0.09; 95% CI, 0.02-0.15), fat mass index (β coefficient, 0.11; 95% CI, 0.04-1.18), BMI (β coefficient, 0.11; 95% CI, 0.05-0.17), and fasting plasma glucose (β coefficient, 0.10; 95% CI, 0.03-0.17) and negatively associated with HDL cholesterol (β coefficient, −0.07; 95% CI, −0.15 to 0.00) (Table 3). Likewise, after further adjusting for the Mediterranean diet score, the associations between 1-SD increments remained significant for the *z* scores of fasting plasma glucose (β coefficient, 0.08; 95% CI, 0.02-0.13) and HDL cholesterol (β coefficient, −0.07; 95% CI, −0.13 to −0.01) (eTable 2 in Supplement 1).

**Table 3.** Association Between Energy-Adjusted Ultraprocessed Food Consumption in Tertiles and 1-SD Increments and Cardiometabolic Risk Factor *z* Scores

Model	Tertiles of energy-adjusted ultraprocessed food consumption, β coefficient (95% CI)			*P* value for trend	Continuous (1-SD increment), β coefficient (95% CI)
	1 (Lowest)	2	3 (Highest)		
**Waist circumference (n = 1390)**					
Crude	1 [Reference]	0.05 (−0.07 to 0.18)	−0.04 (−0.17 to 0.08)	.43	−0.02 (−0.08 to 0.03)
Fully adjusted^a^	1 [Reference]	0.11 (−0.02 to 0.24)	0.20 (0.05 to 0.35)	.01	0.09 (0.02 to 0.15)
**Fat mass index (n = 1219)**					
Crude	1 [Reference]	0.09 (−0.04 to 0.22)	0.16 (0.03 to 0.29)	.02	0.08 (0.03 to 0.14)
Fully adjusted^a^	1 [Reference]	0.13 (−0.00 to 0.27)	0.17 (0.00 to 0.32)	.04	0.11 (0.04 to 1.18)
**Waist-to-height ratio (n = 1389)**					
Crude	1 [Reference]	0.03 (−0.09 to 0.16)	−0.10 (−0.23 to 0.03)	.09	−0.04 (−0.09 to 0.01)
Fully adjusted^a^	1 [Reference]	0.07 (−0.06 to 0.19)	0.12 (−0.03 to 0.27)	.12	0.06 (−0.01 to 0.12)
**BMI (n = 1398)**					
Crude	1 [Reference]	0.12 (−0.01 to 0.24)	0.21 (0.09 to 0.34)	.001	0.10 (0.05 to 0.15)
Fully adjusted^a^	1 [Reference]	0.14 (0.01 to 0.27)	0.20 (0.05 to 0.35)	.009	0.11 (0.05 to 0.17)
**LDL cholesterol (n = 1120)**					
Crude	1 [Reference]	0.03 (−0.11 to 0.17)	−0.13 (−0.27 to 0.01)	.054	−0.05 (−0.11 to 0.00)
Fully adjusted^a^	1 [Reference]	0.01 (−0.13 to 0.16)	−0.06 (−0.23 to 0.11)	.47	−0.03 (−0.09 to 0.04)
**HDL cholesterol (n = 1174)**					
Crude	1 [Reference]	−0.00 (−0.14 to 0.14)	−0.23 (−0.37 to −0.09)	.001	−0.09 (−0.15 to −0.03)
Fully adjusted^a^	1 [Reference]	−0.01 (−0.16 to 0.14)	−0.19 (−0.36 to −0.02)	.02	−0.07 (−0.15 to 0.00)
**Triglycerides (n = 1175)**					
Crude	1 [Reference]	0.09 (−0.05 to 0.23)	0.21 (0.07 to 0.34)	.004	0.07 (0.02 to 0.13)
Fully adjusted^a^	1 [Reference]	0.07 (−0.07 to 0.22)	0.14 (−0.03 to 0.30)	.10	0.04 (−0.03 to 0.11)
**Fasting plasma glucose (n = 1191)**					
Crude	1 [Reference]	0.02 (−0.11 to 0.17)	−0.02 (−0.17 to 0.12)	.69	−0.01 (−0.07 to 0.04)
Fully adjusted^a^	1 [Reference]	0.06 (−0.08 to 0.19)	0.22 (0.06 to 0.37)	.01	0.10 (0.03 to 0.17)
**HOMA-IR (n = 933)**					
Crude	1 [Reference]	0.03 (−0.13 to 0.19)	−0.02 (−0.18 to 0.14)	.78	−0.01 (−0.07 to 0.06)
Fully adjusted^a^	1 [Reference]	0.06 (−0.10 to 0.22)	0.02 (−0.16 to 0.21)	.69	0.02 (−0.06 to 0.10)
**Diastolic blood pressure (n = 1348)**					
Crude	1 [Reference]	0.10 (−0.03 to 0.23)	0.05 (−0.08 to 0.18)	.55	0.01 (−0.04 to 0.07)
Fully adjusted^a^	1 [Reference]	0.08 (−0.05 to 0.21)	−0.01 (−0.16 to 0.14)	.81	−0.01 (−0.08 to 0.05)
**Systolic blood pressure (n = 1346)**					
Crude	1 [Reference]	0.14 (0.01 to 0.27)	0.19 (0.06 to 0.32)	.01	0.08 (0.02 to 0.13)
Fully adjusted^a^	1 [Reference]	0.12 (−0.02 to 0.25)	0.12 (−0.03 to 0.28)	.14	0.05 (−0.01 to 0.11)

Abbreviations: BMI, body mass index; HDL, high-density lipoprotein; HOMA-IR, homeostasis model assessment for insulin resistance; LDL, low-density lipoprotein.

^a^ Fully adjusted for maternal education level, maternal BMI, total minutes of physical activity per week, exclusive breastfeeding, center size, and NOVA classification system groups 1, 2, and 3.

Similar positive associations among fat mass index, BMI, and plasma glucose were observed, irrespective of the animal or vegetable origin of the UPFs consumed. Substitution of 100 g of UPFs with 100 g of unprocessed or minimally processed foods was associated with a decrease in *z* scores of fat mass index (β coefficient, −0.03; 95% CI, −0.06 to 0.00) and BMI (β coefficient, −0.03; 95% CI, −0.06 to −0.01), and fasting plasma glucose (β coefficient, −0.04; 95% CI, −0.07 to −0.01) (Figure). The same models adjusted for the Mediterranean diet score showed positive associations for *z* scores of fasting plasma glucose (β coefficient, −0.04; 95% CI, −0.06 to −0.01) and inverse association for *z* score of HDL cholesterol (β coefficient, 0.03; 95% CI, 0.00-0.07) (eFigure 2 in Supplement 1). No associations were shown for the other outcomes.

**Figure.**
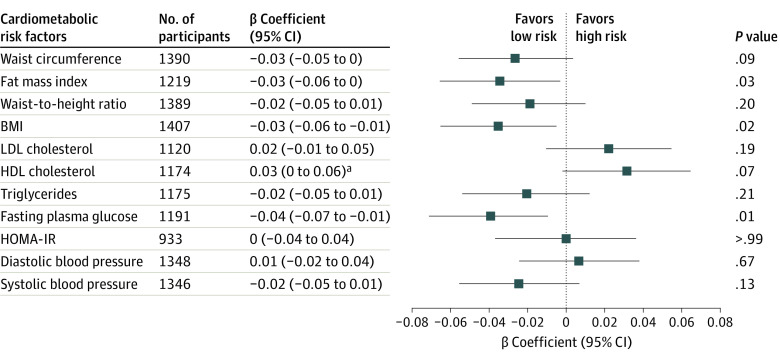
Substitution Analysis of 100 g of Ultraprocessed Food With 100 g of Unprocessed or Minimally Processed Foods and Cardiometabolic Risk Factors Linear regression models were fitted and adjusted for maternal education level, maternal body mass index (BMI), total minutes of physical activity per week, breastfeeding, center size, and NOVA classification system groups 2 and 3. HDL indicates high-density lipoprotein; HOMA-IR, homeostasis model assessment for insulin resistance; LDL, low-density lipoprotein. ^a^For HDL cholesterol, a positive β coefficient signifies low risk.

In children whose mothers were unemployed, a positive association was found between energy-adjusted UPF consumption and *z* scores of waist circumference (β coefficient, 0.26; 95% CI, 0.14-0.39), fat mass index (β coefficient, 0.20; 95% CI, 0.07-0.34), waist-to-height ratio (β coefficient, 0.21; 95% CI, 0.09-0.34), BMI (β coefficient, 0.18; 95% CI, 0.04-0.31), fasting plasma glucose (β coefficient, 0.14; 95% CI, 0.03-0.25), and diastolic blood pressure (β coefficient, 0.14; 95% CI, 0.0-0.27). In children with employed mothers, a positive association was observed between 1-SD increments in energy-adjusted UPF consumption and *z* scores of fasting plasma glucose (β coefficient, 0.09; 95% CI, 0.01-0.17) and a negative association in case of the HDL cholesterol (β coefficient, −0.09; 95% CI, −0.18 to −0.01) (eTable 3 in Supplement 1).

Children whose mothers had a low education level had higher *z* scores of waist circumference (β coefficient, 0.14; 95% CI, 0.05-0.23), fat mass index (β coefficient, 0.15; 95% CI, 0.06-0.25), BMI (β coefficient, 0.15; 95% CI, 0.06-0.24), and fasting plasma glucose (β coefficient, 0.11; 95% CI, 0.02-0.19). Children whose mothers had a high education level had a lower HDL cholesterol *z* score (β coefficient, −0.15; 95% CI, −0.26 to −0.03) (eTable 4 in Supplement 1).

## Discussion

To our knowledge, this study is the first to assess the associations between UPF consumption and various cardiometabolic risk factors in young children. In this large cross-sectional study, UPF consumption was positively associated with *z* scores of BMI, waist circumference, fat mass index, and fasting plasma glucose concentration and inversely associated with HDL cholesterol concentration.

Our findings are in line with those of previous studies.^35,36,37^ Consumption of UPFs at age 4 years was associated with increased BMI *z* scores at age 10 years in the Generation XXI cohort, while no association was found at age 7 years.^35^ Another study showed that high UPF consumption in children aged 7 to 13 years was associated with increased BMI growth trajectories.^36^ Similarly, lower UPF intake in Spanish children aged 4 to 7 years was associated with lower BMI *z* scores at age 7 years, though this association became nonsignificant after adjusting for maternal factors.^37^

A global study by Neri et al^38^ revealed that increased UPF consumption was associated with higher dietary energy density and intake of free sugars, alongside decreased total fiber intake, potentially contributing to childhood obesity. Additionally, findings from the Avon Longitudinal Study of Parents and Children showed that high UPF consumption was associated with unfavorable fat mass index trajectories from age 7 to 24 years.^36^ Similarly, in a Brazilian cohort, UPF consumption during preschool years was associated with increases in waist circumference from preschool to school age.^39^ Other studies found no significant association between UPF consumption and HDL cholesterol and fasting plasma glucose concentrations.^37,40^ Therefore, to our knowledge, our study is the first in children to find significant associations with the aforementioned risk factors and is in line with other studies assessing adult populations.^41^

Our results provide new insight into the association between UPF consumption and health and the importance of recognizing that early dietary habits in childhood might have future implications on cardiometabolic health. While the magnitude of the associations reported in our study may be considered of limited clinical relevance, it is important to note that our study consisted of young children. Therefore, if such minimal differences can reveal a significant association, they may serve as an early warning of future cardiometabolic conditions.

Our results are in line with previous studies showing that the main UPF products consumed are pastries, sweet beverages, cookies, and candies.^42,43,44^ In addition, our results support the findings of other European studies that have shown that children of mothers with lower education or with lower socioeconomic status are more likely to consume UPFs. These findings suggest that educational and socioeconomic factors may contribute to the purchase of low-cost and unhealthy foods, such as UPFs, increasing the risk of health disorders.^37,45,46^

Several possible mechanisms could explain our results. First, UPFs contain higher amounts of sodium, energy, fat, and sugar and lower amounts of fiber, which are well recognized as contributors to cardiometabolic risk factors.^47^ In addition, some UPFs may be linked to a higher glycemic response, and it has been shown that high consumption of sugar-sweetened beverages may delay the internal satiety signal, leading to excessive calorie intake and higher glycemic load.^48,49,50^ Moreover, excessive consumption of energy, saturated fat, and sugar contributes to weight gain and a higher risk of obesity, which is an important risk factor in CVD.^51^ Furthermore, our study showed that children who consumed high amounts of UPFs tended to have lower intakes of fruits and vegetables, which, along with a healthy dietary pattern, are known to be beneficial for cardiometabolic health.^52^

Most of the associations were maintained in our study after further adjusting the models to Mediterranean diet adherence, suggesting that other intrinsic UPF factors may play an important role in determining these associations (eg, additives). Animal and cellular studies revealed potential cardiovascular risks from authorized additives such as sulfites, monosodium glutamate, and emulsifiers.^53,54,55,56,57,58^ Food processing generates contaminants such as acrylamide and acrolein, which have been linked, respectively, to increased odds and risk of cardiovascular disease.^59,60^ Ultraprocessed foods may contain chemicals such as bisphenols and perfluoroalkyl substances that have been associated with a higher risk of cardiometabolic outcomes in children.^61,62^

The NOVA Food Classification system has sparked debate among researchers due to disagreements over UPF definitions, bias concerns, and the system’s contribution to dietary guidelines.^63,64^ The NOVA system itself has some limitations, as it does not consider that certain minimal processing could improve the final product (eg, fermentation in milk) and adopts a vague definition of what is considered a cosmetic additive, which has led to considering carotenoids as an additive that increases the potential harmfulness of a product.^65^ Despite these limitations, NOVA categories have consistently shown associations with cardiometabolic health in adults.

### Strengths and Limitations

This study has several strengths. Most importantly, the study was conducted in a large sample size from 7 different geographic areas of Spain. The study also assessed cardiometabolic risk factors not considered in other similar studies.^21,22,23,24^

Our study also has several limitations. First, because the study is observational, we cannot draw conclusions on cause and effect. Second, our study involved preschool children from Spain, which means that the generalization of our findings to different populations is not appropriate. Third, some grade of misclassification could be present in our study since UPF consumption was estimated from a food and beverage frequency questionnaire that was not specifically developed to assess this type of food, which could result in either an overestimation or underestimation of consumption within various NOVA categories. Additionally, imprecise estimations could also arise from the use of a food and beverage frequency questionnaire, which may be influenced by social desirability bias. Finally, we cannot dismiss that associations may be due to residual confounding or that undetected cardiometabolic disorders in our study population may exist due to age.

## Conclusions

In this large cross-sectional study, UPFs consumption was positively associated with fasting plasma glucose levels, BMI, waist circumference, and fat mass index and inversely associated with HDL cholesterol concentration. These findings highlight the importance of promoting unprocessed or minimally processed foods and reducing UPF consumption, particularly starting from early ages. However, further prospective studies are warranted to validate our findings.

## Notes

Supplement 1. **eAppendix.** Additional Study Assessments
eReference
**eTable 1.** Distribution of FFQ Items Into 4 Groups According to the Degree of Their Processing Established by NOVA Classification System **eFigure 1.** Flow Chart of the Children Included in the Analysis **eTable 2.** Association Between Energy-Adjusted Ultraprocessed Food Consumption in Tertiles (in g/day) and CVD Risk Factors at Baseline **eFigure 2.** Linear Regression Models Replacing 100 g of UPF With 100 g of Unprocessed/Minimally Processed Foods **eTable 3.** Maternal Socioprofessional Stratified Regression Association Between 1-SD Increment of Energy-Adjusted Ultraprocessed Food Consumption (in g/day) and CVD Risk Factors at Baseline **eTable 4.** Maternal Education Level Stratified Regression Association Between 1-SD Increment of Energy-Adjusted Ultraprocessed Food Consumption (in g/day) and CVD Risk Factors at Baseline

Supplement 2.
Data Sharing Statement
