# Supplementary material for: Ultraprocessed Food Consumption and Cardiometabolic Risk Factors in Children
Source: JAMA Netw Open. 2024 May 17;7(5):e2411852. doi: 10.1001/jamanetworkopen.2024.11852 (PMC11102022; doi:10.1001/jamanetworkopen.2024.11852)
Supplement: Supplement 1. — eAppendix. Additional Study Assessments eReference eTable 1. Distribution of FFQ Items Into 4 Groups According to the Degree of Their Processing Established by NOVA Classification System eFigure 1. Flow Chart of the Children Included in the Analysis eTable 2. Association Between Energy-Adjusted Ultraprocessed Food Consumption in Tertiles (in g/day) and CVD Risk Factors at Baseline eFigure 2. Linear Regression Models Replacing 100 g of UPF With 100 g of Unprocessed/Minimally Processed Foods eTable 3. Maternal Socioprofessional Stratified Regression Association Between 1-SD Increment of Energy-Adjusted Ultraprocessed Food Consumption (in g/day) and CVD Risk Factors at Baseline eTable 4. Maternal Education Level Stratified Regression Association Between 1-SD Increment of Energy-Adjusted Ultraprocessed Food Consumption (in g/day) and CVD Risk Factors at Baseline [file jamanetwopen-e2411852-s001.pdf]

Supplemental Online Content

Khoury N, Martínez MÁ, Garcidueñas-Fimbres TE, et al. Ultraprocessed food consumption and cardiometabolic risk factors in children from the CORALS cohort. *JAMA Netw Open*. 2024;7(5):e2411852. doi:10.1001/jamanetworkopen.2024.11852

eAppendix. Additional Study Assessments

eReference

eTable 1. Distribution of FFQ Items Into 4 Groups According to the Degree of Their Processing Established by NOVA Classification System

eFigure 1. Flow Chart of the Children Included in the Analysis

eTable 2. Association Between Energy-Adjusted Ultraprocessed Food Consumption in Tertiles (in g/day) and CVD Risk Factors at Baseline

eFigure 2. Linear Regression Models Replacing 100 g of UPF With 100 g of Unprocessed/Minimally Processed Foods

eTable 3. Maternal Socioprofessional Stratified Regression Association Between 1-SD Increment of Energy-Adjusted Ultraprocessed Food Consumption (in g/day) and CVD Risk Factors at Baseline

eTable 4. Maternal Education Level Stratified Regression Association Between 1-SD Increment of Energy-Adjusted Ultraprocessed Food Consumption (in g/day) and CVD Risk Factors at Baseline

This supplemental material has been provided by the authors to give readers additional information about their work.

© 2024 Khoury N et al. *JAMA Network Open.*

eAppendix. Additional Study Assessments

Dietary intake

Parents or caregivers were asked to indicate, on average, how frequently the participant had consumed the specified serving of each item over the past year, rating their consumption frequency on a scale of 9 options ranging from "never" to "more than 6 times per day ". This questionnaire was designed in machine-readable format to be later scanned using the Evaldara® software. This software efficiently extracts the reported consumption, which was then integrated into the program e-Diet Base URV[1] to obtain the nutrient and energy values.

NOVA classification

The categorization of food items was carried out by two separate dietitians. Additionally, to guarantee precise accuracy, an additional dietitian conducted a thorough revision. Any discrepancies in the classification of particular food items were resolved through extensive discussions among the researchers, ultimately resulting in decisions based on consensus.

Sensitivity analyses

Sensitivity analyses were conducted to assess the potential influence of the nutritional quality of the diet by adjusting for the adherence to Mediterranean diet score. We took into consideration 12 out of 18 points of the score, excluding the points related to UPFs consumption.

Likelihood ratio test was performed to examine the interaction between maternal education level (low education/high education) and socio-professional status (not employed/employed) in relation to the association between UPFs consumption and each of the outcomes. In addition, two stratified analyses by the maternal socio-professional (not employed or employed mothers) and by the maternal education level (low or high education level) were performed to assess potential modifications. Models were adjusted for the aforementioned confounding factors.

eReference

1. QUE ES E-DIETBASE URV? - e-DietBase URV | CeliacBase | SportsBase | DrinkBase. Accessed October 13, 2023. https://e-dieteticaurv.cat/que-es-e-dietbase-urv/

© 2024 Khoury N et al. *JAMA Network Open.*

eTable 1. Distribution of FFQ Items Into 4 Groups According to the Degree of Their Processing Established by NOVA Classification System

**Group 1: Unprocessed or minimally processed foods**	**Milk (whole, semi-skimmed, skimmed), yogurt (without added sugar, skimmed), eggs, chicken (with and without skin), fresh meat (cow, turkey, pork, lamb, rabbit), liver meat, offal meat, fresh fish, oysters, calamari, fresh shrimps, fresh vegetables, gazpacho, fruits, dates, nuts, legumes, rice (white and whole), pasta (white and whole), water, fresh juices, coffee, tea and infusions.**
**Group 2: Processed culinary ingredients**	Olive oil (extra virgin and refined), corn oil, sunflower oil, soybean oil, butter, salt, cacao and sugar.
**Group 3: Processed foods**	Condensed milk, lactose free yogurt, cooking cream, curd, cheeses (white cheese), ham serrano, bacon, salted fish, canned fish, homemade fries, fruits in syrup, olives, bread (white and whole), cereals (with no added sugars and whole) and non-dairy milk.
**Group 4: Ultra-processed food and drink products**	Milkshakes, yogurt (whole and with added sugars), fermented milk, cheese (Petite Suisse, soft cheese), custard, ice cream, ham, processed meat, pate, hamburger, surimi, potato chips, loaf of bread, bread sticks, cereals (with added sugar, flavored-stuffed cereals), pizza, margarine, biscuits (plain, whole, with chocolate), pastries, donuts, muffins, cakes, churros, chocolate, butter cookies, croquettes, ready-to-eat soups, mustard, mayonnaise, ketchup, jams, chocolate cream, snacks, candies, sparkling water, flavored water, bottled juices, soft drinks

Group 1 includes unprocessed or minimally processed foods consisting of natural and whole foods that have undergone little to no processing. Group 2 includes processed culinary ingredients, this group comprises substances extracted from Group 1 foods or nature, used to season and cook foods. Group 3 includes processed food that are manufactured by combining Group 1 or 2 ingredients and may involve some processing methods. Group 4 includes ultra-processed foods (UPF), this group consists of industrial formulations that are characterized by a high energy content, rich in saturated fats, added sugars, salt, with a low amount of fiber and vitamins, which may also contain additives like artificial colors and flavors or stabilizers. Ultra-processed foods are typically ready-to-eat or require minimal preparation (sugary drinks, packaged snacks, fast food, frozen pizzas, and many convenience foods).

© 2024 Khoury N et al. *JAMA Network Open.*

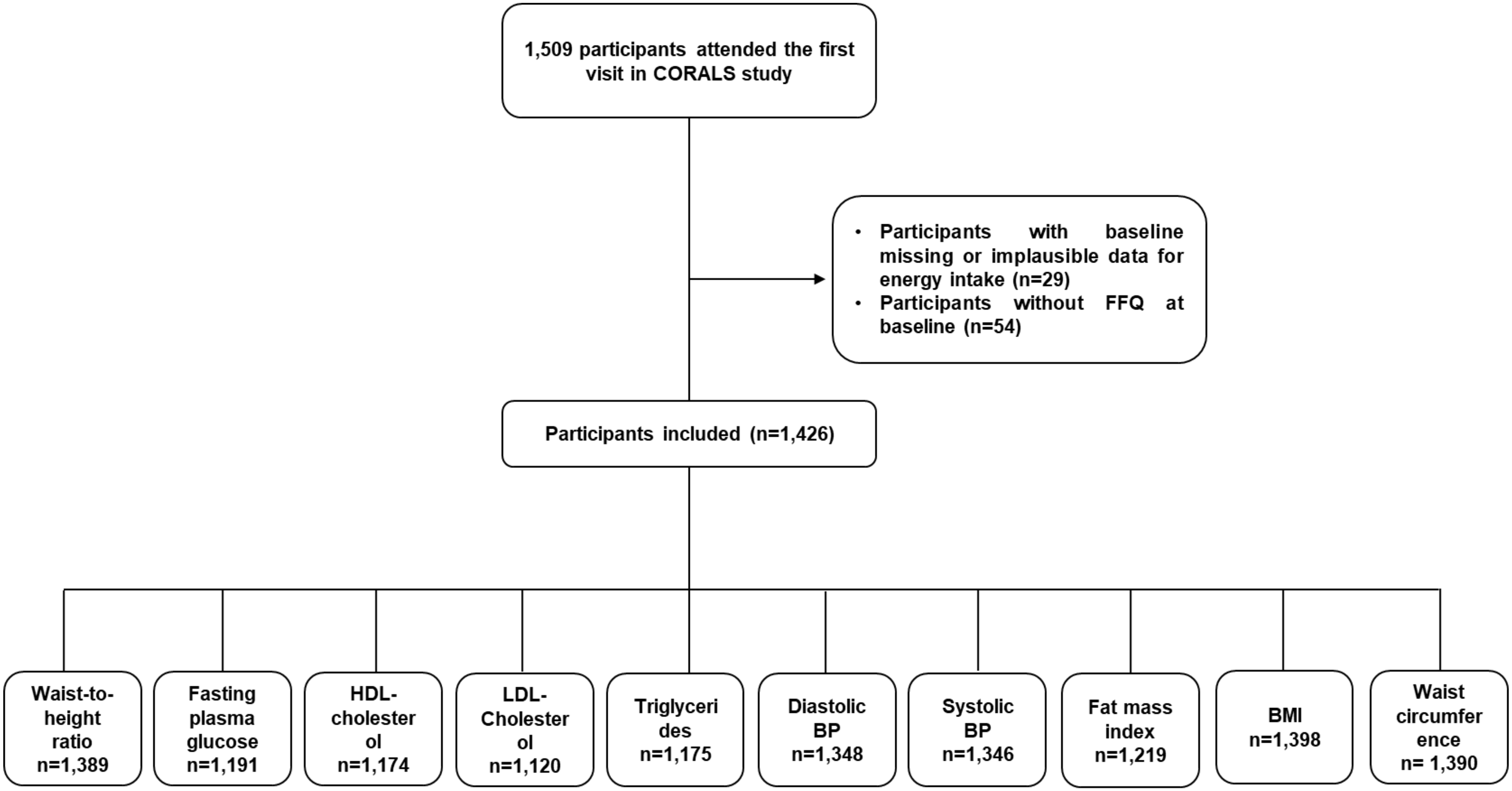

eFigure 1. Flow Chart of the Children Included in the Analysis

© 2024 Khoury N et al. *JAMA Network Open.*

eTable 2. Association Between Energy-Adjusted Ultraprocessed Food Consumption in Tertiles (in g/day) and CVD Risk Factors at Baseline

	**Tertiles of energy-adjusted UPF consumption in g/day β coefficient (95% confidence interval)**				**Continuous (1SD increment)**
	T1	T2	T3	p-trend	
**Waist circumference** (z-score) n= 1,390	0 (ref)	0.04 (-0.09 to 0.17)	0.08 (-0.06 to 0.21)	0.26	0.02 (-0.03 to 0.08)
**Fat mass index** (z-score) n= 1,219	0 (ref)	0.04 (-0.10 to 0.17)	-0.01 (-0.14 to 0.15)	0.97	0.02 (-0.03 to 0.08)
**Waist-to-height ratio** (z-score) n= 1,389	0 (ref)	0.03 (-0.10 to 0.15)	0.05 (-0.08 to 0.18)	0.49	0.02 (-0.03 to 0.07)
**BMI** (z-score) n=1,398	0 (ref)	0.07 (-0.06 to 0.20)	0.08 (-0.05 to 0.22)	0.25	0.04 (-0.01 to 0.09)
**LDL-cholesterol** (z-score) n= 1,120	0 (ref)	0.02 (-0.12 to 0.17)	-0.03 (-0.18 to 0.12)	0.63	-0.01 (-0.07 to 0.05)
**HDL-cholesterol** (z-score) n= 1,174	0 (ref)	-0.03 (-0.18 to 0.12)	**-0.20 (-0.36 to -0.05)**	**0.01**	**-0.07 (-0.13 to -0.01)**
**Triglycerides** (z-score) n= 1,175	0 (ref)	0.04 (-0.10 to 0.18)	0.09 (-0.05 to 0.25)	0.20	0.03 (-0.03 to 0.08)
**Fasting plasma glucose** (z-score) n=1,191	0 (ref)	0.02 (-0.12 to 0.16)	**0.17 (0.03 to 0.31)**	**0.01**	**0.08 (0.02 to 0.13)**
**HOMA-IR** (z-score) n=933	0 (ref)	0.03 (-0.13 to 0.20)	-0.01 (-0.18 to 0.16)	0.86	-0.00 (-0.07 to 0.06)
**Diastolic blood pressure** (z-score) n= 1,348	0 (ref)	0.05 (-0.08 to 0.19)	-0.05 (-0.19 to 0.09)	0.41	-0.03 (-0.08 to 0.03)
**Systolic blood pressure** (z-score) n= 1,346	0 (ref)	0.08 (-0.05 to 0.22)	0.07 (-0.07 to 0.21)	0.36	0.03 (-0.03 to 0.08)

Abbreviations: UPF: ultra-processed food; BMI: Body Mass Index; HDL: High Density Lipoprotein; LDL: Low Density Lipoprotein; HOMA-IR: Homeostatic Model Assessment for Insulin Resistance.

Linear regression models were fitted [β coefficient (95% Confidence Interval)] adjusted for maternal education level, maternal BMI, total minutes of physical activity per week, breastfeeding, center, total energy intake and adherence to Mediterranean diet score (12 points).

© 2024 Khoury N et al. *JAMA Network Open.*

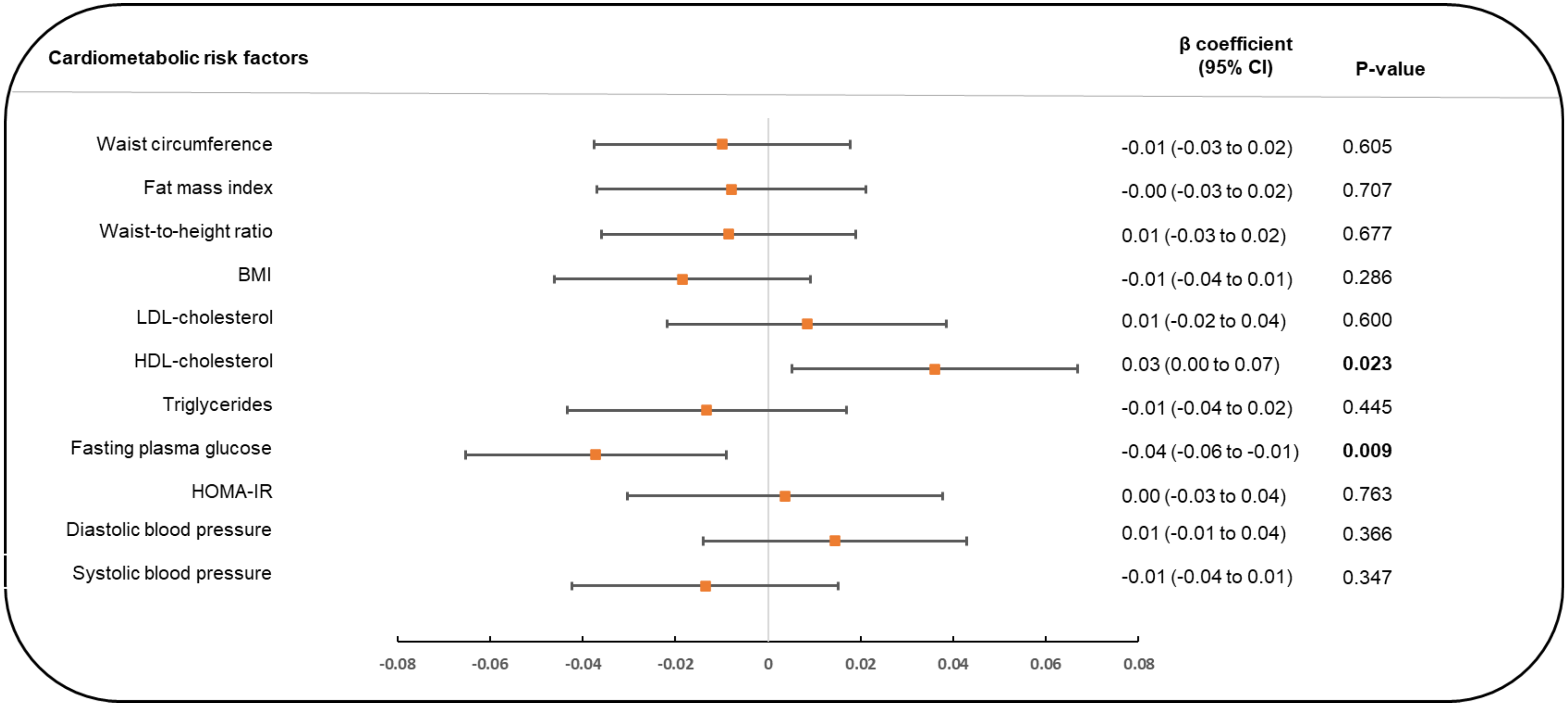

eFigure 2. Linear Regression Models Replacing 100 g of UPF With 100 g of Unprocessed/Minimally Processed Foods

Abbreviations: BMI: Body Mass Index; HDL: High Density Lipoprotein; LDL: Low Density Lipoprotein.
All outcomes are assessed as z-scores
Linear regression models were fitted [β coefficient (95 % Confidence Interval)] adjusted for maternal education level, maternal BMI, total minutes of physical activity per week, breastfeeding, center and adherence to Mediterranean score (12 points)
Fasting plasma glucose n=1,191; HDL-cholesterol n= 1,174; LDL-Cholesterol n= 1,120; Triglycerides n= 1,175; Diastolic BP n= 1,348; Systolic BP n= 1,346; Fat mass index n= 1,219; Waist-to-height ratio n= 1,389; z-BMI n= 1,407; Waist circumference n= 1,390

© 2024 Khoury N et al. *JAMA Network Open.*

eTable 3. Maternal Socioprofessional Stratified Regression Association Between 1-SD Increment of Energy-Adjusted Ultraprocessed Food Consumption (in g/day) and CVD Risk Factors at Baseline

	Energy-adjusted UPF consumption (in g/day, 1SD increment) β coefficient (95% CI)	p-value
**Waist circumference** (z-score)		
Employed (n=1,028)	0.01 (-0.06 to 0.08)	0.80
Not employed (n= 362)	**0.26 (0.14 to 0.39)**	**<0.001**
**Fat mass index** (z-score)		
Employed (n=894)	0.06 (-0.02 to 0.14)	0.12
Not employed (n= 325)	**0.20 (0.07 to 0.34)**	**0.004**
**Waist-to-height ratio** (z-score)		
Employed (n=1,027)	-0.01 (-0.09 to 0.06)	0.70
Not employed (n=362)	**0.21 (0.09 to 0.34)**	**0.001**
**BMI** (z-score)		
Employed (n=1,032)	0.07 (-0.00 to 0.15)	0.05
Not employed (n=366)	**0.18 (0.04 to 0.31)**	**0.01**
**LDL-cholesterol** (z-score)		
Employed (n=815)	-0.05 (-0.13 to 0.04)	0.26
Not employed (n=305)	0.02 (-0.10 to 0.16)	0.70
**HDL-cholesterol** (z-score)		
Employed (n=857)	**-0.09 (-0.18 to -0.01)**	**0.03**
Not employed (n=317)	-0.03 (-0.17 to 0.11)	0.64
**Triglycerides** (z-score)		
Employed (n=858)	0.03 (-0.05 to 0.12)	0.47
Not employed (n=317)	0.08 (-0.04 to 0.20)	0.18
**Fasting plasma glucose** (z-score)		
Employed (n=871)	**0.09 (0.01 to 0.17)**	**0.03**
Not employed (n=320)	**0.14 (0.03 to 0.25)**	**0.01**
**HOMA-IR** (z-score)		
Employed (n=677)	-0.00 (-0.10 to 0.09)	0.96
Not employed (n=256)	0.09 (-0.03 to 0.20)	0.14
**Diastolic blood pressure** (z-score)		
Employed (n=994)	-0.07 (-0.14 to 0.01)	0.09
Not employed (n=354)	**0.14 (0.00 to 0.27)**	**0.04**

© 2024 Khoury N et al. *JAMA Network Open.*

**Systolic blood pressure** (z-score)		
Employed (n=993)	0.02 (-0.06 to 0.10)	0.53
Not employed (n=353)	0.12 (-0.01 to 0.25)	0.07

Abbreviations: BMI: Body Mass Index; HDL: High Density Lipoprotein; LDL: Low Density Lipoprotein.

Linear regression models were fitted [β coefficient (95 % Confidence Interval)] adjusted for maternal education level, maternal BMI, total minutes of physical activity per week, exclusive breastfeeding, center size and NOVA group 1,2 and 3.

Z-score of: Fasting plasma glucose n=1,191; HDL-cholesterol n= 1,174; LDL-Cholesterol n= 1,120; Triglycerides n= 1,175; Diastolic BP n= 1,348; Systolic BP n= 1,346; Fat mass index n= 1,219; Waist-to-height ratio n= 1,389; BMI n= n=1,398; Waist circumference n= 1,390

© 2024 Khoury N et al. *JAMA Network Open.*

eTable 4. Maternal Education Level Stratified Regression Association Between 1-SD Increment of Energy-Adjusted Ultraprocessed Food Consumption (in g/day) and CVD Risk Factors at Baseline

	**Energy-adjusted UPF consumption (in g/day, 1SD increment)**<br>**β coefficient (95% CI)**	**p-value**
Waist circumference (z-score)		
Low education level (n=670)	**0.14 (0.05 to 0.23)**	**<0.01**
High education level (n=720)	0.01 (-0.08 to 0.10)	0.81
Fat mass index (z-score)		
Low education level (n=583)	**0.15 (0.06 to 0.25)**	**<0.01**
High education level (n=636)	0.04 (-0.06 to 0.14)	0.42
Waist-to-height ratio (z-score)		
Low education level (n=669)	0.09 (-0.00 to 0.19)	**0.04**
High education level (n=720)	0.00 (-0.10 to 0.09)	0.99
BMI (z-score)		
Low education level (n=678)	**0.15 (0.06 to 0.24)**	**<0.01**
High education level (n=720)	0.05 (-0.45 to 0.14)	0.28
LDL-cholesterol (z-score)		
Low education level (n=571)	-0.02 (-0.10 to 0.07)	0.69
High education level (n=594)	-0.03 (-0.15 to 0.09)	0.64
HDL-cholesterol (z-score)		
Low education level (n=595)	-0.03 (-0.12 to 0.07)	0.58
High education level (n=579)	**-0.15 (-0.26 to -0.03)**	**0.02**
Triglycerides (z-score)		
Low education level (n=597)	0.05 (-0.04 to 0.14)	0.32
High education level (n=578)	0.02 (-0.09 to 0.13)	0.78
Fasting plasma glucose (z-score)		
Low education level (n=603)	**0.11 (0.02 to 0.19)**	**0.01**
High education level (n=588)	0.09 (-0.01 to 0.20)	0.09
HOMA-IR (z-score)		
Low education level (n=496)	0.03 (-0.10 to 0.15)	0.63
High education level (n=437)	0.01 (-0.08 to 0.09)	0.89
Diastolic blood pressure (z-score)		
Low education level (n=651)	-0.01 (-0.09 to 0.08)	0.89
High education level (n=697)	-0.03 (-0.14 to 0.07)	0.51

© 2024 Khoury N et al. *JAMA Network Open.*

Systolic blood pressure (z-score)		
Low education level (n=650)	0.08 (-0.01 to 0.17)	0.08
High education level (n=696)	0.02 (-0.09 to 0.12)	0.74

Abbreviations: BMI: Body Mass Index; HDL: High Density Lipoprotein; LDL: Low Density Lipoprotein.

Linear regression models were fitted [β coefficient (95 % Confidence Interval)] adjusted for maternal BMI, total minutes of physical activity per week, exclusive breastfeeding, center size and NOVA group 1, 2 and 3.

Z-score of: Fasting plasma glucose n=1,191; HDL-cholesterol n= 1,174; LDL-Cholesterol n= 1,120; Triglycerides n= 1,175; Diastolic BP n= 1,348; Systolic BP n= 1,346; Fat mass index n= 1,219; Waist-to-height ratio n= 1,389; BMI n= n=1,398; Waist circumference n= 1,390

© 2024 Khoury N et al. *JAMA Network Open.*
