# Supplementary material for: Ultraprocessed Food Consumption and Cardiometabolic Risk Factors in Children
Source: JAMA Netw Open. 2024 May 17;7(5):e2411852. doi: 10.1001/jamanetworkopen.2024.11852 (PMC11102022; doi:10.1001/jamanetworkopen.2024.11852)
Supplement: Supplement 2. — Data Sharing Statement [file jamanetwopen-e2411852-s002.pdf]

Data Sharing Statement

Khoury. Ultraprocessed Food Consumption and Cardiometabolic Risk Factors in Children From the CORALS Cohort. *JAMA Netw Open*. Published May 17, 2024. doi:10.1001/jamanetworkopen.2024.11852

Data
Data available: No

Additional Information
Explanation for why data not available: The datasets generated and analyzed during the current study are not publicly available due to data regulations and for ethical reasons, considering that this information might compromise research participants' consent because our participants only gave their consent for the use of their data by the original team of investigators. However, collaboration for data analyses can be requested by sending a letter to the CORALS steering Committee (estudiocoral@corals.es). The request will then be passed to all the members of the CORALS Steering Committee for deliberation.
